# 103 Differences in Estradiol Levels Following Burn Injury

**DOI:** 10.1093/jbcr/irac012.106

**Published:** 2022-03-23

**Authors:** Micah L Willis, Daniel S Soliman, Sally-Irene Ngeve, Cressida Mahung, Shannon Wallet, Robert Maile

**Affiliations:** University of North Carolina Chapel Hill, Durham, North Carolina; University of North Carolina at Chapel Hill, Chapel Hill, North Carolina; North Carolina Central University, Durham, North Carolina; University of North Carolina at Chapel Hill, Washington, District of Columbia; University of North Carolina at Chapel Hill, Chapel Hill, North Carolina; UNC, Chapel Hill, North Carolina

## Abstract

**Introduction:**

The American Burn Association estimates over one million people with burn injuries in the US need medical care, with around 4,500 cases ending in mortality each year. Mortality is due to bacterial infection as a consequence of severe cytokine dysregulation and impaired wound healing. Previous research has shown females have worse outcomes than males following burn injury, but the reasoning is unknown. Estradiol is involved in the regulation of local and systemic interleukin-6 (IL-6) levels, which has been demonstrated by others to positively correlates with poor outcomes following burn. We hypothesize that concentrations of Estrogen can create a pro-inflammatory effect in epithelial cells, which is controlled in a negative feedback loop.

**Methods:**

We utilized plasma from 1) human burn patients and 2) our murine model of burn injury, in which C57BL/6 mice are exposed to a 20% total body surface area burn injury and 3) an *in vitro *cell model using human Oral Epithelial Cells (OECs) and human Airway Epithelial Cells (AECs).

**Results:**

We measured human estradiol levels 1-3 days after burn injury and murine estradiol levels 3 and 7 days after injury by ELISA. In humans there were differences in levels at these timepoints (p< 0.05). In mice we observed a difference in the change in murine estradiol levels between males and females 7 days after injury (p < 0.01). We simulated a wound by removing the cell insert and treating cells with concentrations of estradiol (0.1 nM, 1.0 nM & 250 nM). Images were taken at 0, 6 & 24 H and analyzed using Fiji to observe wound closure. Supernatant was removed from cells at 24 H and analyzed for IL-6 levels (a key pro-inflammatory cytokine linked to poor wound healing after burn) via ELISA. mRNA was isolated from cells and analyzed for IL-6, TNFα & VEGF (important regulators of wound healing) via PCR. We observed an increase in percent wound closure in the AECs, and no difference in OECs. We observed an increase in IL-6 production following stimulation with Estradiol and LPS at different rates for each cell type and corresponding reduction in TNFα and VEGF expression. We also described different Estradiol Receptors in the two cell types.

**Conclusions:**

Taken together, these data suggest burn injury upregulates estradiol in both humans and mice following burn injury whereby this is more robust in females at later stages of burn injury and thus contributing to female-associated poor clinical outcomes.

